# Case of Strangulated Scrotal Hernia

**Published:** 1841-04-03

**Authors:** Thomas F. Betton

**Affiliations:** Germantown


*Case of Strangulated Scrotal Hernia. Operation. Recovery.* By Thomas F. Betton, M. D.

On Monday, the 4th of January, 1841, I was requested by my friend, Dr. Egbert, of Manayunk, to visit with him Mr. Shurr, of that village, who was labouring under strangulated scrotal hernia. The strangulation had occurred on the morning of the previous Friday, at 6 o'clock. The patient did not seek surgical aid until the afternoon of the following Sunday, inasmuch as he had had the affection for forty years, and had always reduced it himself. Dr. Egbert employed the usual means recommended, as well as the tobacco injection, without success, and sent for me at 12 M. on Sunday night. The belladonna was then tried with no better fortune, and the operation alone was left. Owing to the unavoidable absence of his attending physician, it was postponed until 8 o'clock on the following morning. An incision was made nearly the whole length of the scrotum, the cellular tissue, and fasciæ, with the cremaster muscle successively divided, and the sac, which held an unusually large quantity of fluid, exposed. This was divided, and the intestine brought into view. The latter was highly congested, of a deep crimson colour, but otherwise firm and apparently sound. The structure, at the neck of the sac, was divided, and the gut returned to the abdomen. No bad symptoms being present, he was ordered a dose of castor oil, and absolute diet.

*Tuesday,* 9 *A. M.*—Bowels have not been moved, prescribed a large enema, and repeat the oil.

*Evening.*—Still no action of bowels; ordered a pill of calomel gr. ij., opium gr. ½, thrice repeated.

*Wednesday morning.*—Same state; repeat the oil.

*Evening.*—Bowels have been moved freely three or four times; is much better.

*Thursday.*—Ordered calomel gr. ij., ext. hyosciam. gr. i., repeated three times a day; is doing well; bowels gently acted on; wound has adhered throughout a great extent, and looks healthy. Chicken soup, and improved diet. From that time he continued steadily to improve, and is now quite well.

The advanced age of the man, (76 years,) and the length of time of the strangulation, (74 hours,) give the case an interest, in the absence of which I should not claim in your journal the room of more valuable matter.

*Germantown,* March 10th, 1841.

